# Alignments of a Microparticle Pair in a Glow Discharge

**DOI:** 10.3390/molecules26247535

**Published:** 2021-12-13

**Authors:** Evgeny A. Lisin, Evgeny A. Kononov, Eduard A. Sametov, Mikhail M. Vasiliev, Oleg F. Petrov

**Affiliations:** 1 Joint Institute for High Temperatures, Russian Academy of Sciences, 125412 Moscow, Russia; gadvin@yandex.ru (E.A.K.); vasiliev@ihed.ras.ru (M.M.V.); ofpetrov@ihed.ras.ru (O.F.P.); 2 Moscow Institute of Physics and Technology, National Research University, 141700 Dolgoprudny, Russia

**Keywords:** active matter, colloids, dusty plasma, stability, gas discharge

## Abstract

Stability of a vertically aligned microparticle pair in a stratified glow DC discharge is experimentally investigated. Using laser perturbations, it is shown that, for the same discharge parameters, a pair of microparticles can be suspended in two stable configurations: vertical and horizontal. The interparticle interaction and the electric field of the stratum in the region of particle levitation are quantitatively investigated for the first time. The decharging effect of the lower (downstream) particle by the ion flow wake is also observed for the first time in a glow discharge. The obtained experimental data made it possible to check the analytical criteria for the configurational stability of the system.

## 1. Introduction

A colloidal plasma is a nontrivial instance of soft condensed matter [1]. Under certain conditions, the colloidal plasma becomes a thermodynamically open, non-Hamiltonian system that can exist in liquid-like or crystal-like states [2,3] and exhibit the properties of active matter [4,5,6]. When micron-sized particles are immersed in a gas discharge with ion flow, they acquire significant negative charges (typically, 10^3^–10^4^ elementary charges) and can create ion wakes by the flow of ions past particles [7,8,9]. The resulting “particle–particle” interaction is complex and effectively nonreciprocal: a microparticle experiences an electrostatic repulsion from the like-charged adjacent particles and an effective attraction to their ion wakes. Due to such wake-mediated interparticle interaction, the like-charged microparticles suspended in a capacitively coupled radio-frequency (RF) discharge can form vertical pairs, despite significant vertical compression by the electric field of the sheath [10,11,12]. The stability of the vertical configuration increases with a decharging of the lower (downstream) particle by the ion wake [13,14,15,16] and in the presence of strong horizontal interaction with neighboring particles forming extended bilayered and multi-layered crystals [10,11,17,18]. In some experiments, particles of different masses were used to assemble their vertical configuration [19,20,21,22]. Single vertical particle pairs and chain-like structures were also created by applying an additional horizontal confinement [21,23,24,25,26,27,28]. Single vertical chains consisting of several dozen microparticles were also observed in an induction RF discharge and a glow DC discharge [29,30,31].

The possible coupling of two identical particles and the stability of their alignment in the electric field of a gas discharge were theoretically investigated in Refs. [12,16,32,33,34]. The problem of breaking the chain-like configuration of a finite number of particles due to the lateral instability onset was considered in Refs. [35,36,37]. Nevertheless, the obtained analytical criteria for the configuration stability of various systems have never been compared with experimental data.

Here, the stability of a vertically aligned microparticle pair in a stratified glow DC discharge is experimentally investigated. The interparticle interaction and the electric field of the stratum in the region of particle levitation are quantitatively investigated for the first time. For this purpose, we use a recently developed method [27] based on the analysis of the spectral density of random processes. Unlike most studies, this method does not require any external disturbances and a special design of the experimental setup, preliminary measurements of external fields and assumptions about the type of interaction. A brief history of previous experimental studies of nonreciprocal interaction forces between particles in a colloidal plasma and a description of the methods used for this are given in Ref. [27]. The obtained experimental data made it possible to check the analytical criteria for the configurational stability of the system.

## 2. Criteria for Particle Stability

Consider a system of two interacting particles in a glow discharge stratum. The gas-discharge tube is installed vertically in a gravity field directed opposite to the Z axis. For definiteness, we assign the index “1” to the lower particle, i.e., located lower along the Z axis, and “2” to the upper one. The gravity and electrostatic repulsion of two particles are compensated by the confining electric field of the stratum. The particles are considered to be strongly coupled thus they are in thermal motion near their equilibrium positions.

Consider the lateral stability of the system, i.e., resistance to small displacements of particles perpendicular to the line connecting the equilibrium positions of the particles. The stability of the vertical configuration is determined by the response of the system to displacements in the horizontal plane (for example, along the X axis), and to determine the stability of the horizontal configuration, displacements of particles only in the vertical direction (along the Z axis) are considered. If the condition [16,38]

(1) $$\frac{f_{21}^{\left(\xi \right)}}{\left|f_{1}^{\left(\xi \right)}\right|}+\frac{f_{12}^{\left(\xi \right)}}{\left|f_{2}^{\left(\xi \right)}\right|}<1$$

is met, then the configuration is stable. Here $f_{ij}^{\left(\xi \right)}$ is the stiffness of the ξ-component of the specific force $F_{ij}/M$ acting on the *j*-th particle with mass $M_{j}$ from the *i*-th one, $f_{j}^{\left(\xi \right)}$ is the stiffness of the ξ-component of the specific confining force $F_{j}/M_{j}$ acting on the *j*-th particle from the stratum. The selection of the force component and the derivative direction indicates by the superscript: ξ≡x or z.

We further assume that the particles have equal mass, $M_{j}\equiv M$, carry different negative charges ($Q_{1}$ < 0 and $Q_{2}$ < 0), and the electric field strength E(r,z) is linear with a radial component $E_{r}=\alpha r$ and a vertical component $E_{z}=E_{0}+\beta z$, where $r\equiv {\left(x^{2}+y^{2}\right)}^{1/2}$ is the radial coordinate, z. is the vertical coordinate, α > 0 and β > 0 are the gradients of the electric field strength, and $E_{0}$ is determined by the balance of forces acting in the system. Earlier experiments [14,15,27] showed that with a vertical arrangement of particles (parallel to the ion flow), the charge of the lower (downstream) particle becomes less than the charge of the upper particle due to the decharging effect caused by the ion wake, i.e., |$Q_{1}$|<|$Q_{2}$|. For a horizontally aligned particle pair (perpendicular to the ion flow), the charges of the particles are equal ($Q_{1}=Q_{2}\equiv Q$), since in this case the levitation height of the particle depends on the ratio of charge to mass.

If the vector of the interparticle interaction force $F_{ij}$ and the vector of the interparticle distance $r_{ij}=r_{i}-r_{j}$ are collinear, then, taking into account the force balance equation [16,33,35], the condition (1) is reduced to:

(2) $$\frac{\alpha^{*}}{\beta }>1.\;$$

for the vertical configuration of particles, where

(3) $$\alpha^{*}=\alpha +\left(\frac{1}{\left|Q_{1}\right|}-\frac{1}{\left|Q_{2}\right|}\right)\frac{Mg}{L},$$

g is the acceleration of gravity, and $L=r_{ij}$ is the separation of the particles in equilibrium; and

(4) $$\frac{\alpha }{\beta }<1$$

for the horizontal configuration. Note that the collinearity of $F_{ij}$ and $r_{ij}$ holds for the entire family of potentials with a spherical symmetry, the simplest example of which is the Coulomb potential. Obviously, for particles with equal charges, the condition (2) is reduced to α/β >1 [35].

## 3. Experiment

An experimental study was carried out in a stratified glow DC discharge. A gas-discharge glass tube with a length of 1250 mm and an inner diameter of 40 mm was evacuated to an operating pressure of 2 Pa. After that, a direct current discharge was ignited between the anode and cathode, the distance between which was 1050 mm. During the experiment, the discharge current increased from 0.75 mA to 1.95 mA. In the upper part of the tube, there was a microparticle injector in the form of a container with a piezoelectric emitter. The container was filled with monodisperse polystyrene particles with a diameter of 4.6 μm, which were thrown into the discharge one by one when a signal from a pulse generator was applied to the piezoelectric emitter. The particles fell into the region of the positive column of the discharge, where they acquired an electric charge and were captured by strata. The experiments were carried out with both two and three microparticles in different spatial configurations in the lower stratum. Microparticles were visualized using a homogeneously expanded beam (with a diameter of 3 cm) of a 200 mW solid-state laser, passed through the glass bottom of the tube. The movement of particles was recorded in the vertical plane using a high-speed video camera with a recording frequency of 200 frames per second and a spatial resolution of 215 pixels per mm. Using a computer video processing, the particle trajectories were determined. Further spectral analysis of the trajectories of two interacting particles made it possible to determine the directional derivatives of the interparticle interaction forces and the external confining forces with which the stratum acts on the particles. In order to determine the ratio of the gradients of the stratum field components in the region of particle levitation, an additional analysis of the dynamics of a solitary particle in the stratum was carried out at the same discharge parameters that had been set in experiments with two particles.

A second solid-state laser with a power of up to 1.5 watts was used to manipulate the particles by the radiation pressure of the focused laser beam (1 mm in diameter). Note that similar manipulations with a particle pair levitating in a RF discharge were carried out earlier [14,19,24,26].

**Figure 1:**
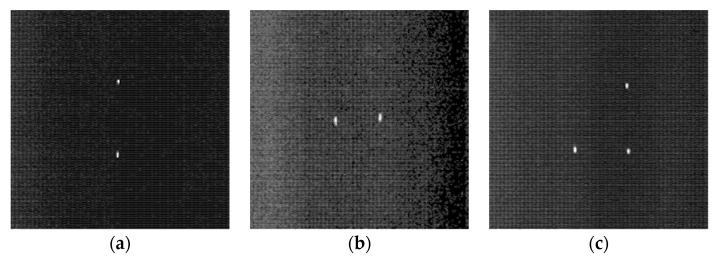
Video frames of different particle configurations in the lower stratum of a glow DC discharge at a buffer gas pressure of 2 Pa and a current of 0.75 mA. (**a**) Vertical alignment of two particles before the laser perturbation (we assign the index “1” to the lower particle and “2” to the upper one); (**b**) particles in the horizontal plane after the laser action on the lower particle; (**c**) stable configuration of three particles.

When two microparticles were injected into the stratum of the gas discharge tube, they were always located one above the other along the vertical axis of the tube (see Figure 1a). Due to the action of the laser pulse, the lower particle was kicked aside, and the vertical pair changed its alignment to horizontal. When the laser exposure was stopped, the horizontal configuration was retained (see Figure 1b). Interestingly, when the laser pulse hit the upper particle, the configuration did not change. Thus, laser manipulations have shown that for the same discharge parameters, a pair of microparticles can be suspended in two stable configurations: vertical and horizontal. The observed response of the vertically aligned particle pair to laser impact gives reason to suggest that with an increase in the oscillation amplitude of the lower particle, an amplitude instability may occur with a subsequent transition to a horizontal configuration. One can assume that such a situation is possible, for example, when using light-absorbing particles [39,40] or Janus particles [5,41,42], i.e., active particles, the kinetic energy of which increases with an increase in the laser illumination intensity. Note that when an additional third particle was injected, the coexistence of vertical and horizontal configurations was observed, see Figure 1c.

## 4. Results and Discussion

### 4.1. Forces Acting on the Particles

**Figure 2:**
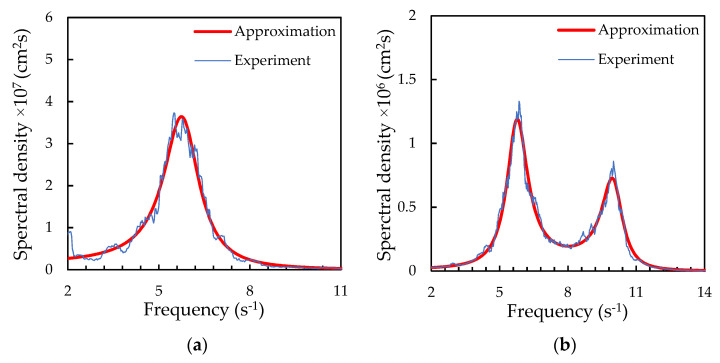
Spectral density of horizontal oscillations of (**a**) the solitary particle and (**b**) the lower particle in the system of two vertically aligned particles. The thin line shows the experimental data obtained for 1.05 mA, and the bold line plots the fit by the analytical function [27].

**Figure 3:**
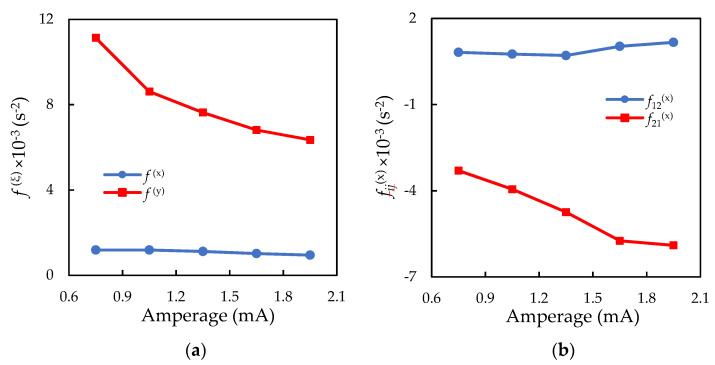
Stiffness of specific (**a**) confining forces acting on the solitary particle ($f^{\left(x\right)}$ and $f^{\left(z\right)}$) and (**b**) interaction forces acting between the vertically aligned particles ($f_{21}^{\left(x\right)}$ and $f_{12}^{\left(x\right)}$), depending on the discharge current.

**Figure 4:**
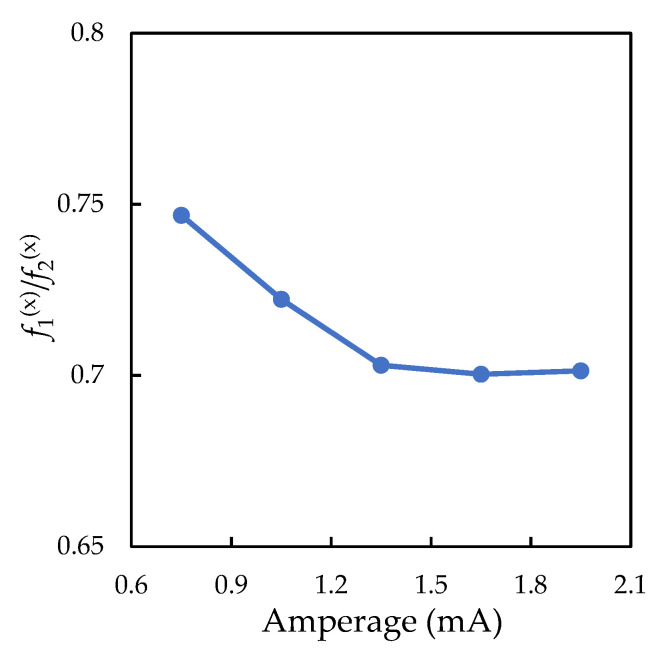
Ratio of $f_{1}^{\left(x\right)}/f_{2}^{\left(x\right)}$ for the vertically aligned particles, depending on the discharge current.

Using the method proposed in [27], a spectral analysis of the particle trajectories was carried out. Figure 2 shows examples of the oscillation spectra of a solitary particle and a vertically aligned particle pair. The approximation of the obtained spectral densities for a solitary particle provides information on the forces acting on the charged microparticle from the stratum, namely: $f^{\left(x\right)}=\alpha Q/M$ and $f^{\left(z\right)}=\beta Q/M$. The approximation of the spectral densities for the vertically aligned particle pair made it possible to determine the stiffness of specific interaction forces ($f_{21}^{\left(x\right)}$ and $f_{12}^{\left(x\right)}$) and confining forces ($f_{1}^{\left(x\right)}$ and $f_{2}^{\left(x\right)}$). Figure 2 shows how analytical functions fit the experimentally measured spectral densities of the oscillations of a solitary particle and a vertically aligned particle pair. Experimental data on the forces acting on the solitary particle ($f^{\left(x\right)}\;\mathrm{and}\;f^{\left(z\right)}$) and between the vertically aligned particles ($f_{21}^{\left(x\right)}$ and $f_{12}^{\left(x\right)}$) are presented in Figure 3 in dependence on the discharge current strength. The ratio of $f_{1}^{\left(x\right)}/f_{2}^{\left(x\right)}$ for the vertical particle pair is shown in Figure 4. The statistical error in measuring the forces, caused by fitting the experimental spectral density with some noise, can be estimated by “bootstrapping” [43]. The confidence intervals, which contain 95% of the bootstrapped values, are within ±8% for the confining forces and ±11% for the interparticle interaction forces.

The stratum thickness was about 3 cm at all currents. The particles levitated approximately three millimeters below the stratum center. With increasing discharge current, the average separation of the vertically aligned particles decreases from 0.7 to 0.4 mm. A rough estimate of the ion Debye length, based on the available numerical data [44,45], gives the same order of magnitude as the particle separation. Since the particles were located far from the stratum edge and the particle separation was much smaller than the stratum thickness, then, for further analysis of the experimental results, we assume that the plasma conditions in the vicinity of the particles are relatively constant. By analogy with Ref. [27], for monodisperse particles we have

(5) $$f_{1}^{\left(x\right)}/f_{2}^{\left(x\right)}\approx Q_{1}/Q_{2}.$$

Figure 4 shows with an increase in the discharge current, the $Q_{1}/Q_{2}$ ratio changed from 0.75 to 0.7. As the voltage increases, the speed of the drifted ions increases, and the upstream particle amplifies the ion flows to the downstream negatively charged particle. This result is in agreement with previous experiments carried out in capacitive RF discharges [14,15,27,46], as well as with numerical simulations [13,47], exhibiting what has been called the decharging effect. Interestingly, $f^{\left(x\right)}$ and $f_{2}^{\left(x\right)}$ values, obtained for a single particle and the upper particle of a vertical pair, respectively, are practically the same, taking into account measurement errors. The same result was previously observed in a RF discharge [15].

### 4.2. Experimental Verification of the Criteria for Particle Stability

**Figure 5:**
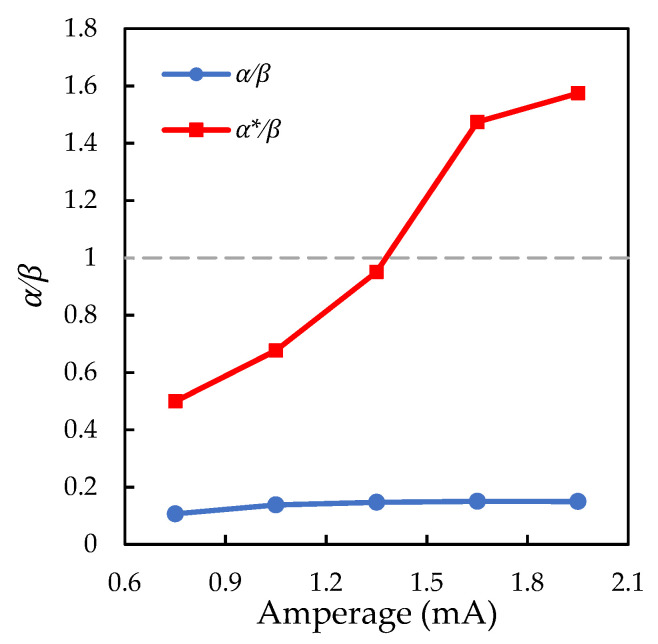
Ratio of the horizontal to vertical electric field gradient α/β and the ratio $\alpha^{*}/\beta$ included in the left part of the inequality (2), depending on the discharge current.

The obtained experimental data make it possible to check the analytical criteria for the configurational stability of the system. The experiment with a solitary microparticle shows that over the entire range of discharge current (from 0.75 mA to 1.95 mA) at a pressure of 2 Pa, the electric field gradient along the gas discharge tube axis (vertical axis) is several times higher than the radial (horizontal) gradient at the area of particle levitation, see the blue curve in Figure 5. Under these conditions, when the particles have equal masses, charges and symmetrical interaction, then according to the condition (4), horizontal alignment is preferable for them. In our experiments, with the horizontal alignment of two particles, their charges can be considered equal, and an effective violation of the interparticle interaction symmetry can be neglected.

When α/β < 1, as follows from the conditions (1) and (2), for the lateral stability of the vertical configuration of two identical particles, in addition to the external confinement, the following conditions are required: unequal charges of particles ($Q_{1}/Q_{2}$ < 1) and an effective breaking of the interparticle interaction symmetry ($f_{21}^{\left(x\right)}\neq f_{12}^{\left(x\right)}$). Such inequalities can arise in the presence of ion drift caused by an axial electric field.

**Figure 6:**
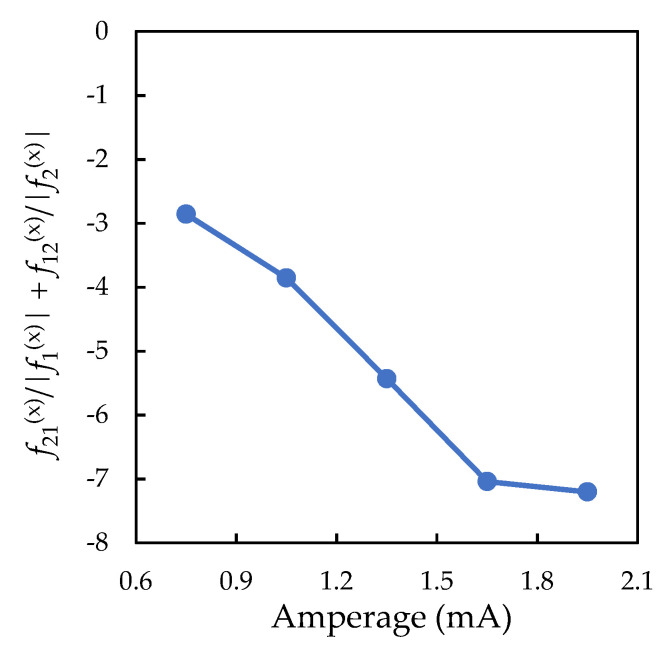
Left part of the inequality (1), depending on the discharge current.

Taking into account Equation (3) for $\alpha^{*}$ and Equation (5) for $Q_{1}/Q_{2}$ ratio, the left part of inequality (2) can be rewritten as $\alpha^{*}/\beta =\left\{\left|f_{2}^{\left(x\right)}\right|+\left(f_{2}^{\left(x\right)}/f_{1}^{\left(x\right)}-1\right)g/L\right\}/\left|f^{\left(z\right)}\right|$. Since the condition (2) is not satisfied at a current strength of less than 1.4 mA (see the red curve in Figure 5), the stability of the vertical pair must be additionally ensured by the inequality of the derivatives of the interparticle interaction forces. Figure 6 shows the left part of the inequality (1) depending on the discharge current strength. It is easy to see that the condition (1) for a vertically aligned particle pair holds for all discharge currents.

## 5. Conclusions

The stability of a vertically aligned microparticle pair in a stratified glow DC discharge has been experimentally investigated. Using laser perturbations, we have shown that, for the same discharge parameters, a pair of microparticles can be suspended in two stable configurations: vertical and horizontal. The interparticle interaction and the electric field of the stratum in the region of particle levitation have been quantitatively investigated for the first time. The decharging effect of the lower (downstream) particle by the ion flow wake was also observed for the first time in a glow discharge. Using the experimental data, the analytical criteria for the configuration stability of the system were verified. Note that these criteria can be used to describe the configuration stability of two particles with very different nonreciprocal interactions. Examples include flowing colloidal suspensions where the nonreciprocity may occur due to depletion forces [48,49,50,51,52] acting on closely spaced macroparticles moving through a colloidal dispersion.

## Data Availability

The data are available from the corresponding authors upon reasonable request.
